# NIK is required for NF-*κ*B-mediated induction of BAG3 upon inhibition of constitutive protein degradation pathways

**DOI:** 10.1038/cddis.2014.584

**Published:** 2015-03-12

**Authors:** F Rapino, B A Abhari, M Jung, S Fulda

**Affiliations:** 1 Institute for Experimental Cancer Research in Pediatrics, Goethe-University, Frankfurt, Germany; 2 Institute of Pharmaceutical Sciences, Albert-Ludwigs-University, Freiburg, Germany; 3 German Cancer Consortium (DKTK), Heidelberg, Germany; 4 German Cancer Research Center (DKFZ), Heidelberg, Germany

## Abstract

Recently, we reported that induction of the co-chaperone Bcl-2-associated athanogene 3 (BAG3) is critical for recovery of rhabdomyosarcoma (RMS) cells after proteotoxic stress upon inhibition of the two constitutive protein degradation pathways, that is, the ubiquitin-proteasome system by Bortezomib and the aggresome-autophagy system by histone deacetylase 6 (HDAC6) inhibitor ST80. In the present study, we investigated the molecular mechanisms mediating BAG3 induction under these conditions. Here, we identify nuclear factor-kappa B (NF-*κ*B)-inducing kinase (NIK) as a key mediator of ST80/Bortezomib-stimulated NF-*κ*B activation and transcriptional upregulation of BAG3. ST80/Bortezomib cotreatment upregulates mRNA and protein expression of NIK, which is accompanied by an initial increase in histone H3 acetylation. Importantly, NIK silencing by siRNA abolishes NF-*κ*B activation and BAG3 induction by ST80/Bortezomib. Furthermore, ST80/Bortezomib cotreatment stimulates NF-*κ*B transcriptional activity and upregulates NF-*κ*B target genes. Genetic inhibition of NF-*κ*B by overexpression of dominant-negative I*κ*B*α* superrepressor (I*κ*B*α*-SR) or by knockdown of p65 blocks the ST80/Bortezomib-stimulated upregulation of BAG3 mRNA and protein expression. Interestingly, inhibition of lysosomal activity by Bafilomycin A1 inhibits ST80/Bortezomib-stimulated I*κ*B*α* degradation, NF-*κ*B activation and BAG3 upregulation, indicating that I*κ*B*α* is degraded via the lysosome in the presence of Bortezomib. Thus, by demonstrating a critical role of NIK in mediating NF-*κ*B activation and BAG3 induction upon ST80/Bortezomib cotreatment, our study provides novel insights into mechanisms of resistance to proteotoxic stress in RMS.

Regulation of protein quality control (PQC) is critical to prevent proteotoxicity caused by accumulation of misfolded or insoluble proteins.^1^ In mammalian cells there are both constitutive and inducible PQC systems. Under basal conditions, PQC is maintained by the ubiquitin-proteasome system (UPS) and the aggresome-autophagy system. The latter involves the cytoplasmic histone deacetylase 6 (HDAC6) that promotes dynein-dependent retrograde transport of protein aggregates along microtubules to form aggresomes and mediates autophagosome-lysosome fusion.^2^ When constitutive PQC mechanisms are impaired, Bcl-2-associated athanogene 3 (BAG3)-dependent selective autophagy can be engaged as an inducible compensatory mechanism to avoid proteotoxicity.^3^ BAG3 is a co-chaperone that promotes the recognition of misfolded proteins, their transport to the microtubule-organizing center to form aggresomes and their removal via selective autophagy.^3^

Nuclear factor-kappa B (NF-*κ*B) is a key transcription factor of the cellular stress response in cancer.^4^ Two main NF-*κ*B pathways have been described, that is, the canonical and the non-canonical NF-*κ*B signaling pathways.^5^ The canonical NF-*κ*B pathway is activated by various cytokines, for example, tumor necrosis factor (TNF)*α*, which, upon its binding to the TNF receptor (TNFR), stimulates the phosphorylation and activation of IKKβ within the I*κ*B kinase (IKK) complex. IKKβ in turn phosphorylates I*κ*B*α*, thereby prompting its ubiquitination and subsequent degradation via the proteasome. This releases the NF-*κ*B proteins p65 (RelA) and p50 to translocate into the nucleus where they transactivate NF-*κ*B target genes.

In the non-canonical NF-*κ*B pathway, NF-*κ*B-inducing kinase (NIK) is constitutively degraded under resting conditions via a multiprotein complex containing cellular inhibitor of apoptosis (cIAP)1/2 and TNF receptor-associated factor (TRAF)2/3.^6^ Ligation of TNFR family members such as CD40 causes the ubiquitination and subsequent degradation of cIAP proteins, which in turn terminates this constitutive degradation of NIK, leading to NIK accumulation and activation of IKK*α*. Once activated, IKK*α* phosphorylates the NF-*κ*B precursor protein p100, which initiates its partial proteasomal processing to generate the p52 NF-*κ*B subunit. p52 then translocates to the nucleus to activate NF-*κ*B target genes. In addition to these post-translational mechanisms, there is mounting evidence showing that NIK expression is regulated at the transcriptional level. Epigenetic events including histone acetylation have recently been reported to govern the expression of NIK in human cancers.^7^ Disruption of a closed chromatin structure by HDAC inhibitors has been described to lead to enhanced NIK expression and NF-*κ*B activation.^7^ There exist several crosstalks between the canonical and the non-canonical NF-*κ*B pathway.^5^ For example, NIK phosphorylates not only IKK*α* but also IKKβ, thereby activating both the non-canonical and canonical NF-*κ*B pathway.^6^ Moreover, NIK has been reported to phosphorylate p65, which enhances its transcriptional activity.^8^

In a heat shock model of proteotoxic stress, activation of NF-*κ*B has been shown to be required for the removal of aggregated damaged proteins via the induction of BAG3.^9^ We recently showed that simultaneous inhibition of two constitutive PQC pathways, that is, the UPS by Bortezomib and the aggresome-autophagy pathway by the cytoplasmic HDAC6 inhibitor ST80, stimulates BAG3 expression in rhabdomyosarcoma (RMS) cells that survive the cotreatment and are able to regrow after drug removal.^10^ BAG3 induction is critically required to mitigate ST80/Bortezomib-triggered proteotoxicity by clearing protein aggregates via selective autophagy.^10^ However, little is yet known about the molecular mechanisms that are involved in the regulation of BAG3 expression under conditions of proteotoxicity. Therefore, we investigated the underlying mechanisms responsible for BAG3 induction upon proteotoxic stress in the present study.

## Results

### ST80/Bortezomib cotreatment induces NF-*κ*B activation

**Figure 1:**
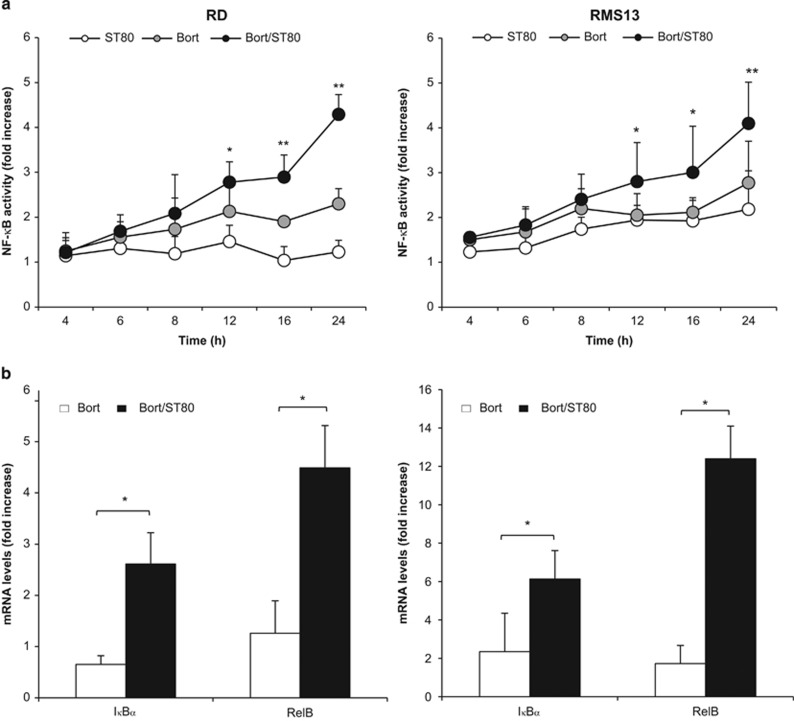
NF-*κ*B is activated upon ST80/Bortezomib cotreatment. (**a**) RMS cells stably transfected with pTRH1- NF-*κ*B EGFP plasmid were treated with 20 nM (RD) or 50 nM (RMS13) Bortezomib and/or 50 *μ*M ST80 at indicated time points. NF-*κ*B activation was measured by flow cytometry. Data are shown as fold increase of GFP compared with the untreated cells. (**b**) RMS cells were treated with 20 nM (RD) or 50 nM (RMS13) Bortezomib and/or 50 *μ*M ST80 for 12 h. RelB and I*κ*B*α* mRNA levels were quantified by RT-PCR. Mean+S.D. of three independent experiments performed in triplicate are shown; *P<0.05; **P<0.01

Recently, we showed that the co-chaperone BAG3 is transcriptionally upregulated in RMS cells that survive concomitant inhibition of the two major constitutive protein degradation pathways, that is, the UPS and the aggresome/autophagy pathway, by cotreatment with the proteasome inhibitor Bortezomib and the HDAC6 inhibitor ST80.^10^ Furthermore, we demonstrated that BAG3 has a pivotal role in mediating cell recovery upon ST80/Bortezomib cotreatment by promoting the clearance of cytotoxic protein aggregates via selective autophagy.^10^ Since NF-*κ*B represents a key transcription factor that controls the cellular stress response,^4^ we asked whether NF-*κ*B has a role in BAG3 induction upon ST80/Bortezomib cotreatment. To address this question we monitored NF-*κ*B activation in RMS cell lines of both the embryonal and alveolar subtype, that is, RD and RMS13 cells, respectively. To this end, we created RMS cells with stable expression of a green fluorescent protein (GFP)-labeled NF-*κ*B reporter construct and analyzed the kinetics of NF-*κ*B activation. Intriguingly, ST80/Bortezomib cotreatment significantly enhanced NF-*κ*B activity compared with cells treated with either agent alone (Figure 1a). Stimulation with the prototypic NF-*κ*B stimulus TNF*α* was used as the positive control (Supplementary Figure S1). Furthermore, ST80/Bortezomib cotreatment significantly increased mRNA levels of I*κ*B*α* and RelB, two known NF-*κ*B target genes, compared with cells treated with Bortezomib alone (Figure 1b), confirming that ST80/Bortezomib cotreatment triggers NF-*κ*B activation.

### NF-*κ*B is required for ST80/Bortezomib-stimulated upregulation of BAG3

**Figure 2:**
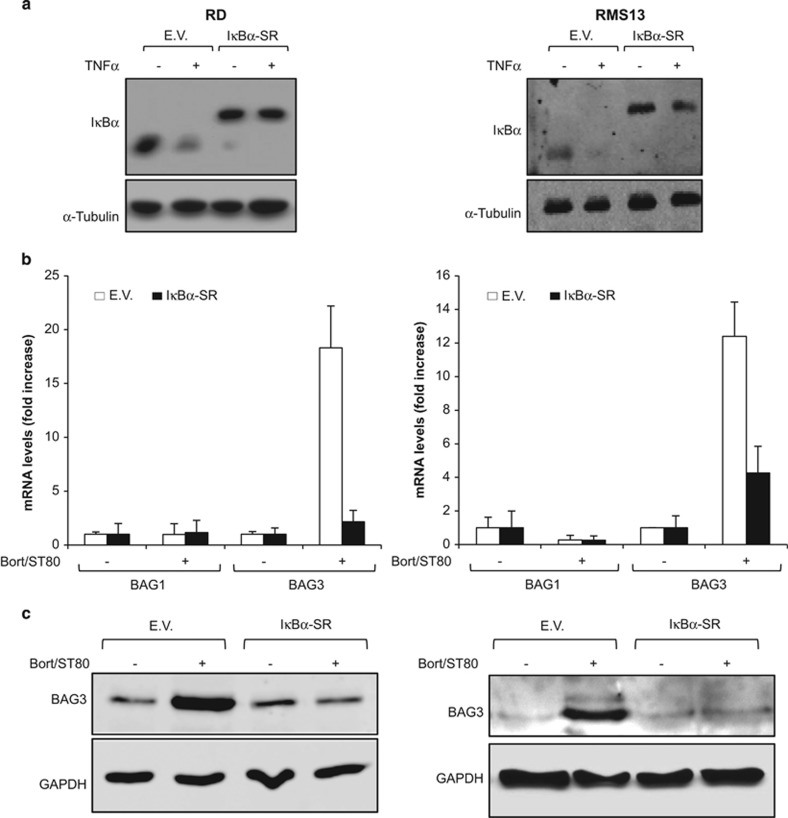

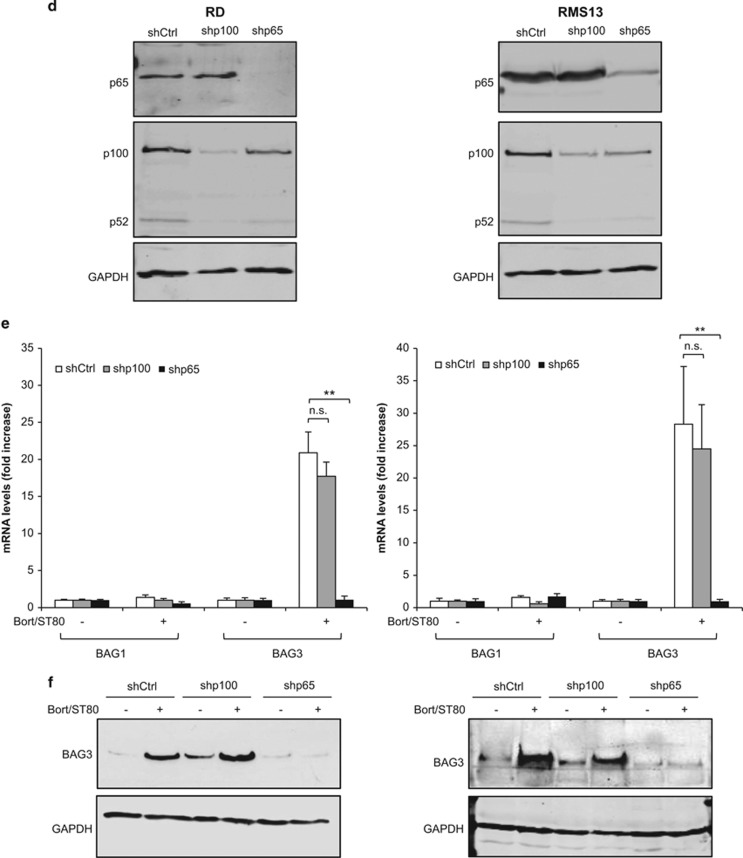
ST80/Bortezomib-surviving cells upregulate BAG3 in an NF-*κ*B dependent manner. (**a**–**c**) RMS cells were stably transfected with pCFG5-IEGZ vector (E.V.) or pCFG5-IEGZ vector containing I*κ*B*α*-S(32, 36)A (I*κ*B*α*-SR). In (**a**), transfection efficiency was assessed by western blot analysis after treatment with 10 ng/ml TNF*α* for 3 h. *α*-Tubulin was used as loading control. In (**b** and **c**), cells were treated with 20 nM (RD) or 50 nM (RMS13) Bortezomib and 50 *μ*M ST80 for 48 h. BAG1 and BAG3 mRNA levels were assessed by RT-PCR (**b**); BAG3 protein levels were assessed by Western blot analysis. GAPDH was used as loading control (**c**). (**d**–**f**) RMS cells transduced with control vector (shCtrl) or vectors containing shRNA sequence against p65 or p100 were treated with 20 nM (RD) or 50 nM (RMS13) Bortezomib and 50 *μ*M ST80 for 48 h. Transduction efficiency was assessed by western blot analysis. GAPDH was used as the loading control (**d**). BAG1 and BAG3 mRNA levels were assessed by RT-PCR (**e**); BAG3 protein levels were assessed by western blot analysis. GAPDH was used as loading control (**f**). In (**b** and **e**), mean+S.D. of three independent experiments performed in triplicate are shown; n.s., non significant; **P<0.01

To investigate the question whether NF-*κ*B activation is required for ST80/Bortezomib-stimulated upregulation of BAG3, we overexpressed a dominant-negative phosphomutant of I*κ*B*α*, that is, I*κ*B*α* superrepressor (I*κ*B*α*-SR), which is insensitive to proteasomal degradation upon NF-*κ*B stimulation by TNF*α* (Figure 2a). Control experiments confirmed that transcriptional activation of the prototypic NF-*κ*B target gene TNF*α* was blocked in I*κ*B*α*-SR cells compared with the empty vector cells (Supplementary Figure S2a). Interestingly, NF-*κ*B inhibition by I*κ*B*α*-SR profoundly impaired the induction of BAG3 mRNA as well as protein levels upon ST80/Bortezomib cotreatment (Figures 2b and c), pointing to a key role of NF-*κ*B in BAG3 upregulation. In parallel, we determined mRNA levels of the co-chaperone BAG1 to test the specificity of NF-*κ*B-mediated upregulation of BAG3 by ST80/Bortezomib (Figure 2b). In contrast to its effect on BAG3, ST80/Bortezomib cotreatment did not increase BAG1 mRNA expression (Figure 2b), underlining that ST80/Bortezomib cotreatment selectively upregulates BAG3 levels.

To confirm the involvement of NF-*κ*B in BAG3 upregulation, we also generated RMS cells with stable knockdown of p65 (shp65) and p100 (shp100), key components of the canonical and non-canonical NF-*κ*B pathway, respectively (Figure 2d). As control, we used an RNA sequence with no corresponding counterpart in the human genome (Figure 2d). p65 knockdown cells were incapable to upregulate TNF*α* mRNA levels upon NF-*κ*B stimulation by TNF*α* (Supplementary Figure S2b), demonstrating that NF-*κ*B activation was blocked in these cells. In addition, p65 knockdown cells exhibited reduced p100 protein levels (Figure 2d), consistent with p100 being a known NF-*κ*B target gene.^11^ Importantly, p65 knockdown prevented upregulation of BAG3 mRNA and protein levels after ST80/Bortezomib treatment compared with control cells (Figures 2e and f). By comparison, p100 knockdown had little effects on ST80/Bortezomib-stimulated upregulation of BAG3 (Figures 2e and f). Consistently, p100 knockdown cells exhibited a similar transcriptional upregulation of TNF*α* as control cells (Supplementary Figure S2b), demonstrating that p100 silencing was not able to prevent ST80/Bortezomib-stimulated NF-*κ*B activation. In contrast to BAG3, no differences in BAG1 mRNA levels were detected upon ST80/Bortezomib cotreatment in p65 or p100 knockdown cells (Figure 2e), confirming the specificity of ST80/Bortezomib-stimulated upregulation of BAG3. Taken together, this set of experiments shows that NF-*κ*B is required for BAG3 induction upon ST80/Bortezomib cotreatment in RMS cells.

### ST80/Bortezomib cotreatment upregulates NIK mRNA and protein expression

**Figure 3:**
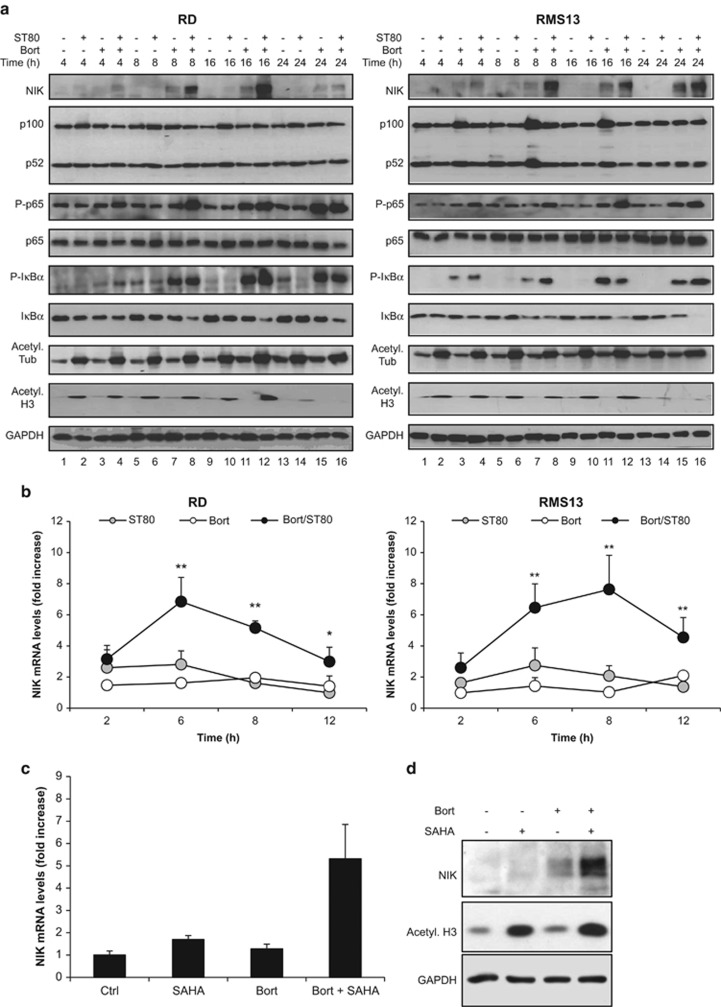
ST80/Bortezomib stimulates canonical NF-*κ*B activation. (**a** and **b**) RMS cells were treated with 20 nM (RD) or 50 nM (RMS13) Bortezomib and/or 50 *μ*M ST80 at indicated time points. NF-*κ*B key regulatory proteins levels were assessed by western blot analysis. GAPDH was used as loading control (**a**). NIK mRNA levels were measured by RT-PCR (**b**). (**c**) RD were treated with 20 nM Bortezomib and/or 1 *μ*M SAHA for 6 h. NIK mRNA levels were measured by RT-PCR. (**d**) RD were treated with 20 nM Bortezomib and/or 1 *μ*M SAHA for 8 h. Histone H3 acetylation and NIK protein levels were assessed by western blot analysis. GAPDH was used as loading control. In (**b** and **c**), mean+S.D. of three independent experiments performed in triplicate are shown; *P<0.05; **P<0.01

To explore the mechanisms involved in ST80/Bortezomib-stimulated NF-*κ*B activation, we monitored expression levels and/or phosphorylation status of major components of the canonical and non-canonical NF-*κ*B pathways by western blot analysis. Interestingly, we found that ST80/Bortezomib cotreatment caused marked upregulation of NIK protein levels (Figure 3a lines 8, 12, 16). Also, ST80/Bortezomib cotreatment increased phosphorylation of p65 and I*κ*B*α* and reduced I*κ*B*α* levels, in line with the activation of the canonical NF-*κ*B pathway (Figure 3a lines 4, 8, 12 and 16). We did not find convincing evidence for processing of p100 protein to p52 upon ST80/Bortezomib cotreatment (Figure 3a), which might be due to inhibition of proteasomal processing of p100 in the presence of Bortezomib.

Since we found that NIK expression is markedly increased by ST80/Bortezomib cotreatment compared with treatment with Bortezomib alone, we next asked whether NIK upregulation is controlled by transcriptional mechanisms. Since transcriptional upregulation of NIK has recently been linked to increased histone H3 acetylation,^7^ we analyzed the acetylation status of histone H3. Importantly, treatment with ST80 or ST80/Bortezomib transiently increased acetylation of histone H3 at early time points up to 16 h, in parallel with accumulation of NIK (Figure 3a). In contrast, acetylation of *α*-Tubulin, a known target of HDAC6, remained elevated over an extended period of time in cells exposed to ST80 or ST80/Bortezomib (Figure 3a). These findings indicate that ST80 transiently increases acetylation of histone H3, while it causes prolonged acetylation of *α*-Tubulin.

In order to explore whether the ST80-stimulated acetylation of histone H3 leads to transcriptional upregulation of NIK, we quantified NIK mRNA levels. Notably, ST80/Bortezomib cotreatment significantly increased NIK mRNA levels with peak induction at 6–8 h after cotreatment (Figure 3b), in line with the kinetics of NIK protein upregulation (Figure 3a). To further explore the relevance of histone H3 acetylation for NIK induction, we used suberoylanilide hydroxamic acid (SAHA) as another HDAC inhibitor.^12^ Similarly, SAHA/Bortezomib cotreatment upregulated NIK mRNA and protein levels (Figures 3c and d). Taken together, these data show that ST80/Bortezomib cotreatment triggers upregulation of NIK mRNA and protein expression.

### NIK is required for ST80/Bortezomib-mediated NF-*κ*B activation and BAG3 induction

**Figure 4:**
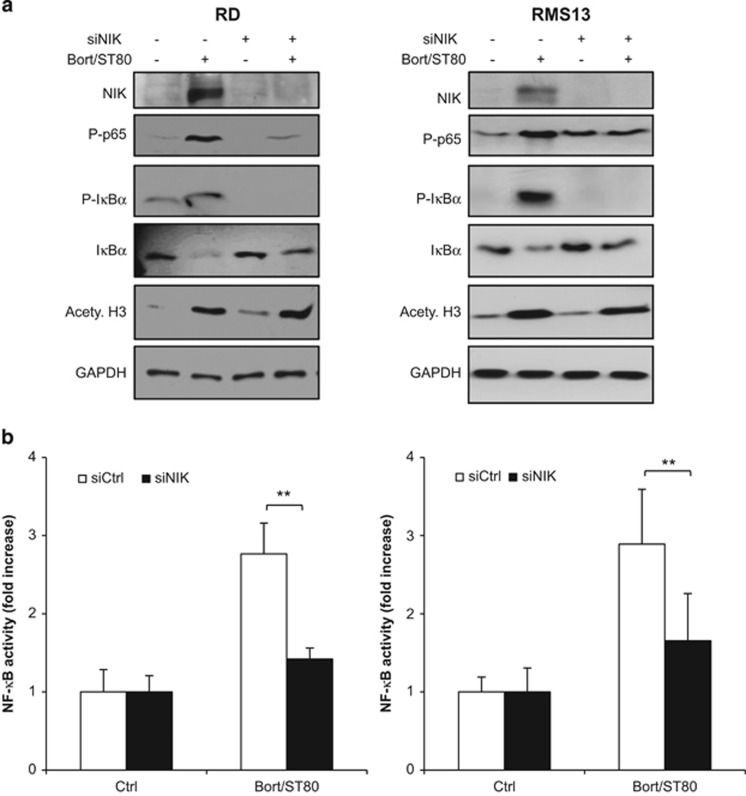

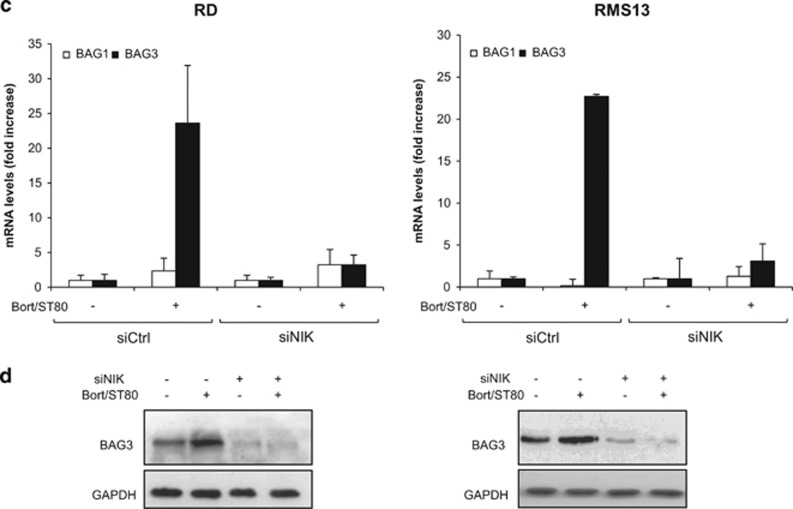
NIK mediates NF-*κ*B activity and BAG3 transcription. RMS cells stably transfected with pTRH1- NF-*κ*B EGFP plasmid were transiently transfected with siRNA control sequence (siCtrl) or siRNA sequence against NIK (siNIK). (**a**) Cells were treated with 20 nM (RD) or 50 nM (RMS13) Bortezomib and 50 *μ*M ST80 for 8 h. NF-*κ*B key regulatory proteins levels were assessed by western blot analysis. GAPDH was used as loading control. (**b**) Cells were treated with 20 nM (RD) or 50 nM (RMS13) Bortezomib and 50 *μ*M ST80 for 16 h (RD) or 24 h (RMS13). NF-*κ*B activation was measured by flow cytometry. Data are shown as fold increase of GFP compared with the untreated cells. (**c** and **d**) Cells were treated with 20 nM (RD) or 50 nM (RMS13) Bortezomib and 50 *μ*M ST80 for 48 h. BAG1 and BAG3 mRNA levels were assessed by RT-PCR (**c**). BAG3 protein levels were assessed by western blot analysis. GAPDH was used as loading control (**d**). In (**b**) and (**c**), mean+S.D. of three independent experiments performed in triplicate are shown; **P<0.01

To determine the functional requirement of NIK, we transiently knocked down NIK by siRNA. NIK silencing attenuated phosphorylation of p65 and I*κ*B*α* as well as degradation of I*κ*B*α* upon ST80/Bortezomib cotreatment, while it did not interfere with acetylation of H3 (Figure 4a and Supplementary Figure S3), suggesting that NIK is involved in the activation of the canonical NF-*κ*B pathway. In addition, knockdown of NIK significantly reduced ST80/Bortezomib-stimulated NF-*κ*B transcriptional activation in RMS cells expressing a GFP-labeled NF-*κ*B reporter plasmid compared with cells transfected with control siRNA (Figure 4b).

Next, we investigated the role of NIK in regulating BAG3 induction by ST80/Bortezomib. Importantly, NIK knockdown abolished the ST80/Bortezomib-triggered upregulation of BAG3 mRNA and protein levels compared with control cells (Figures 4c and d), in line with inhibition of NF-*κ*B activation by NIK knockdown (Figures 4a and b). Additionally, NIK silencing reduced constitutive expression of BAG3 protein in RMS cells (Figure 4d). In contrast, BAG1 mRNA levels were not affected by NIK silencing confirming the specificity of NIK-mediated upregulation of BAG3 (Figure 4c). Taken together, these results demonstrate that NIK is a critical mediator of NF-*κ*B activation and subsequent upregulation of BAG3 upon ST80/Bortezomib treatment.

### Lysosomal activity is necessary for ST80/Bortezomib-mediated NF-*κ*B activation and BAG3 induction

**Figure 5:**
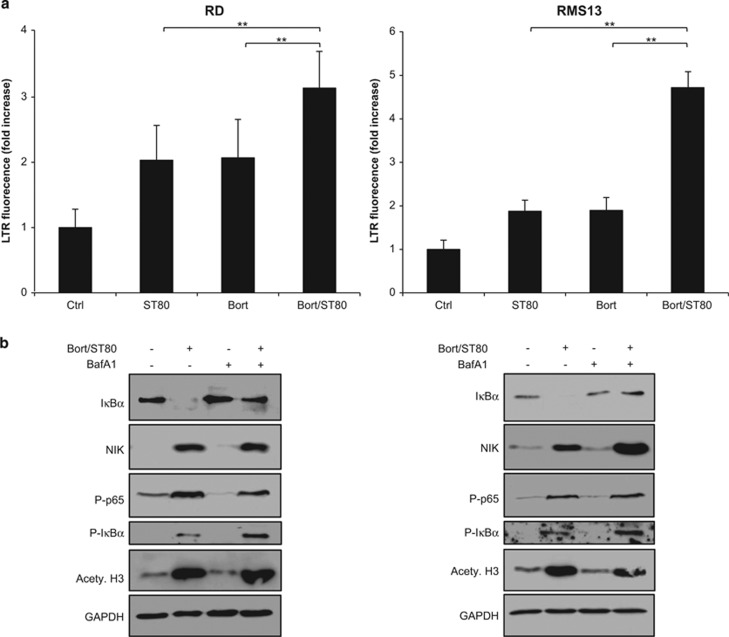

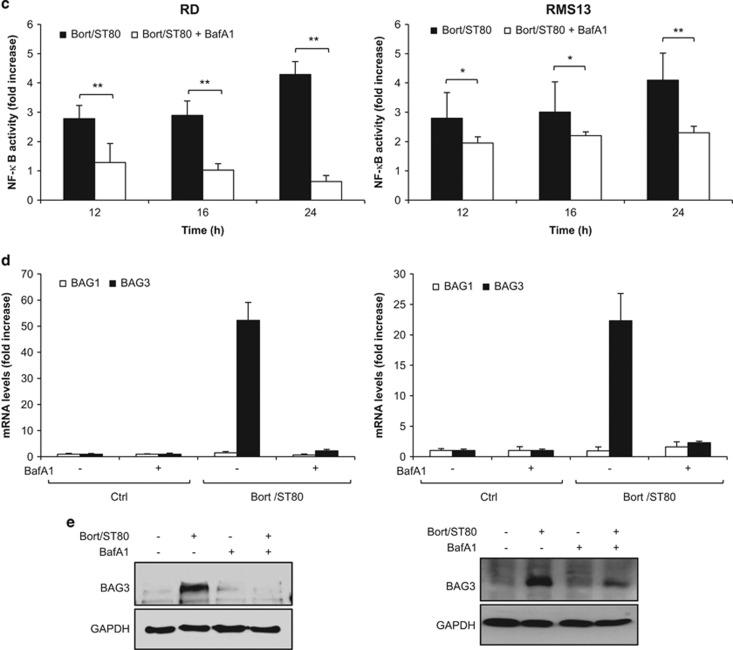
I*κ*B*α* degradation is mediated by lysosomes upon ST80/Bortezomib cotreatment. (**a**) RMS cells were treated with 20 nM (RD) or 50 nM (RMS13) Bortezomib and 50 *μ*M ST80 for 8 h. Lysosomal acidification was quantified by FACS measurement of Lysotracker Red stained cells. (**b**) RMS cells were treated with 20 nM (RD) or 50 nM (RMS13) Bortezomib and 50 *μ*M ST80 in the presence and absence of 10 nM BafA1 for 8 h. NF-*κ*B key regulatory proteins levels were assessed by western blot analysis. GAPDH was used as loading control. (**c**) RMS cells stably transfected with pTRH1- NF-*κ*B EGFP plasmid were treated with 20 nM (RD) or 50 nM (RMS13) Bortezomib and 50 *μ*M ST80 in the presence or absence of 10 nM BafA1 at indicated time points. NF-*κ*B activation was measured by FACS analysis of FITC florescence. Data are shown as fold increase of GFP compared with untreated cells. (**d** and **e**) RMS cells were treated with 20 nM (RD) or 50 nM (RMS13) Bortezomib and 50 *μ*M ST80 in the presence and absence of 10 nM BafA1 for 48 h. BAG1 and BAG3 mRNA levels were assessed by RT-PCR (**d**). BAG3 protein levels were assessed by western blot analysis. GAPDH was used as loading control (**e**). In (**a**, **c** and **d**), mean+S.D. of three independent experiments performed in triplicate are shown; *P<0.05; **P<0.01

Since we observed that ST80/Bortezomib cotreatment triggers the degradation of I*κ*B*α* (Figure 3a), we next asked how I*κ*B*α* is degraded when the proteasome is inhibited by Bortezomib. Since the lysosomal compartment has been implicated in the degradation of key components of the NF-*κ*B signaling pathway,^13^ we hypothesized that I*κ*B*α* degradation occurs via the lysosomal route. To test this hypothesis, we quantified lysosomal activity by Lysotracker Red staining. Of note, ST80/Bortezomib cotreatment significantly increased lysosomal activity compared to either compound alone (Figure 5a). To explore whether lysosomal degradation is responsible for I*κ*B*α* degradation and subsequent NF-*κ*B activation following ST80/Bortezomib cotreatment, we monitored NF-*κ*B activation in the presence and absence of Bafilomycin A1 (BafA1), an inhibitor of vacuolar H^+^ ATPases that inhibits lysosomal degradation.^14^ Intriguingly, addition of BafA1 attenuated the ST80/Bortezomib-triggered degradation of I*κ*B*α* protein, whereas it did not block NIK accumulation, phosphorylation of I*κ*B*α* and p65 or acetylation of histone H3 (Figure 5b). Furthermore, addition of BafA1 significantly impaired ST80/Bortezomib-stimulated NF-*κ*B activation at all tested time points, whereas BafA1 did not alter constitutive NF-*κ*B activity in untreated cells (Figure 5c and Supplementary Figure S4a). Importantly, addition of BafA1 prevented the ST80/Bortezomib-stimulated upregulation of BAG3 mRNA and protein levels, whereas it had no effect on BAG1 mRNA levels (Figures 5d and e). Also, BafA1 blocked the ST80/Bortezomib-stimulated transcriptional induction of other prototypic NF-*κ*B target genes such as I*κ*B*α* and RelB (Supplementary Figure S4b), confirming that inhibition of lysosomal degradation by BafA1 blocks the ST80/Bortezomib-mediated transcriptional activation of NF-*κ*B.

To investigate whether macroautophagy is responsible for the delivery of I*κ*B*α* to lysosomes for degradation, we knocked down ATG5 by siRNA. Silencing of ATG5 did not prevent Bort/ST80-mediated downregulation of I*κ*B*α* (Supplementary Figure S5), suggesting that macroautophagy is not essential for lysosomal degradation of I*κ*B*α*. Together, this set of experiments indicates that I*κ*B*α* is degraded via the lysosome upon ST80/Bortezomib cotreatment, which in turn leads to NF-*κ*B transcriptional activation and BAG3 upregulation.

## Discussion

**Figure 6:**
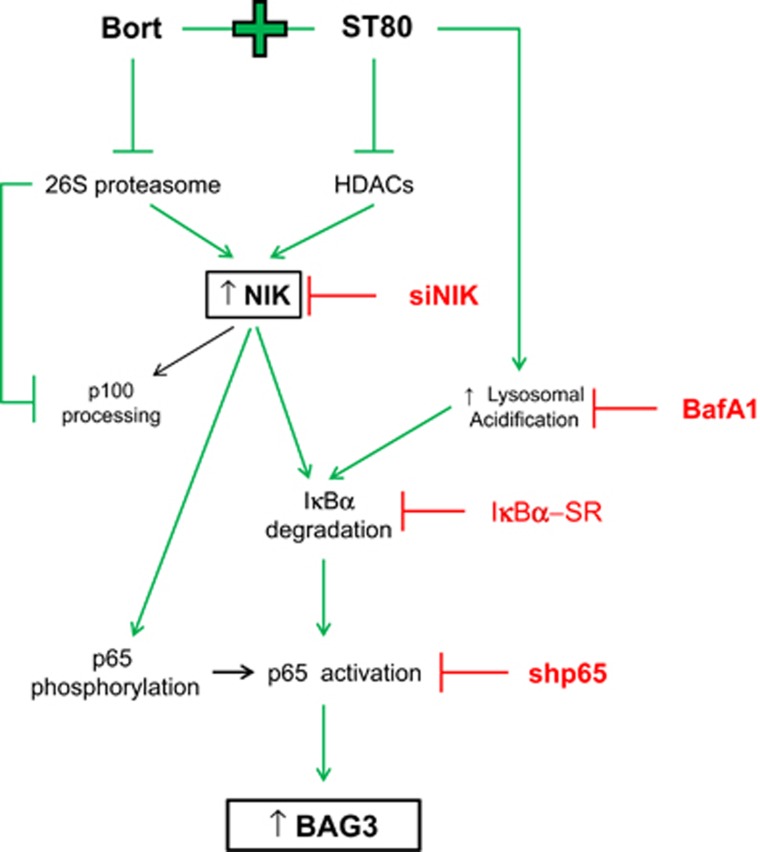
Scheme of the proposed mechanism. Bortezomib and ST80 cotreatment leads to NIK transcriptional induction and protein accumulation which in turns triggers the activation of the NF-*κ*B canonical signaling pathway and finally BAG3 upregulation (green lines). See text for details

Inducible resistance in response to drug treatment is one of the major limitations in cancer therapy. Recently, we discovered that concomitant inhibition of the two constitutive protein degradation pathways, that is, the UPS by Bortezomib and the aggresome-autophagy system by the HDAC6 inhibitor ST80, stimulates upregulation of the co-chaperone BAG3, which is critical to support the survival and recovery of RMS cells following ST80/Bortezomib treatment by promoting the clearance of protein aggregates through selective autophagy.^10^ In the present study, we therefore investigated the underlying molecular mechanisms that are responsible for ST80/Bortezomib-stimulated upregulation of BAG3. Here, we identify NIK as a key player of NF-*κ*B-mediated upregulation of BAG3 upon ST80/Bortezomib cotreatment. We propose a model where ST80/Bortezomib cotreatment increases NIK mRNA and protein levels, which leads to NF-*κ*B activation and transcriptional upregulation of BAG3 (Figure 6). Several lines of evidence support this conclusion. First, ST80-mediated acetylation of histone H3 and Bortezomib-imposed blockage of proteasomal degradation cooperate to increase both mRNA and protein levels of NIK. Second, ST80/Bortezomib cotreatment triggers phosphorylation of p65 and I*κ*B*α*, degradation of I*κ*B*α*, NF-*κ*B transcriptional activation and upregulation of NF-*κ*B target genes. Third, genetic silencing of NIK prevents phosphorylation of p65 and I*κ*B*α*, NF-*κ*B activation and BAG3 induction upon ST80/Bortezomib cotreatment, underscoring the crucial role of NIK in NF-*κ*B-mediated upregulation of BAG3. Fourth, NF-*κ*B inhibition either by overexpression of I*κ*B*α*-SR or knockdown of p65 abolishes the ST80/Bortezomib-stimulated upregulation of BAG3.

The novelty of the present study resides in the discovery that NIK is a critical mediator of BAG3 induction by ST80/Bortezomib. Cotreatment with ST80/Bortezomib triggers the accumulation of NIK mRNA and protein levels. In addition to the proposed mode of action of the HDAC6 inhibitor ST80 to cause sustained acetylation of *α*-Tubulin as a prototypical substrate of HDAC6, we found in the present study that ST80 also transiently increases histone H3 acetylation. Nevertheless, cotreatment with ST80/Bortezomib is required to substantially upregulate NIK mRNA and protein expression.

Post-translational regulation of NIK protein expression has been extensively studied in recent years, as NIK is a short-lived protein that is subject to constitutive proteasomal degradation.^6^ In line with this notion, inhibition of the proteasome has been described to cause accumulation of NIK protein levels.^15^ By comparison, little is yet known about transcriptional control of NIK expression. Recently, elevated NIK expression has been linked to increased histone H3 acetylation of NIK promoter in basal-like breast cancer.^7^ In addition, treatment with the pan-HDAC inhibitor valproic acid was shown to upregulate NIK expression, suggesting that chromatin opening by histone H3 acetylation promotes NIK transcription.^7^ Also, glucocorticoids have been reported to transcriptionally upregulate NIK via a glucocorticoid response element (GRE) motif within the NIK promoter.^16^ Of note, NIK has been characterized as a kinase that activates both canonical and non-canonical NF-*κ*B pathways.^6^ Besides stimulating p100 processing, which engages non-canonical NF-*κ*B signaling, NIK has been implicated in phosphorylating components of the canonical NF-*κ*B pathway including I*κ*B*α* and p65.^6, 8^ Consistently, we demonstrate that NIK is required for phosphorylation of I*κ*B*α* and p65 in ST80/Bortezomib-cotreated cells, since knockdown of NIK abrogates these phosphorylation events.

Induction of NF-*κ*B has previously been shown to be important for cell recovery from heat shock via upregulation of a BAG3-HspB8 complex, which led to removal of aggregated and damaged proteins.^9, 17^ Nevertheless, the specific signaling events responsible for NF-*κ*B-mediated transcription of BAG3 have remained elusive. Although the BAG3 promoter harbors a putative NF-*κ*B consensus site, no direct binding of NF-*κ*B to BAG3 promoter region was detected by ChIP assay after heat shock.^17^ Our data showing that ST80/Bortezomib-stimulated NF-*κ*B activation occurs early within the first 12–16 h upon drug removal, while BAG3 is upregulated later after 32–36 h,^10^ also point to an indirect transcriptional induction of BAG3 by NF-*κ*B. Further, BAG3 has been reported to be transcriptionally regulated by heat shock factor 1 (HSF1), a transcription factor that enhances cancer cell survival under various forms of stress.^18, 19^ Thus, additional studies are required to elucidate in detail the signaling events involved in BAG3 induction upon proteotoxicity.

Another interesting finding of our work is that I*κ*B*α*, a cytoplasmic inhibitor of NF-*κ*B that is usually processed via the proteasome in the course of NF-*κ*B activation,^4^ is degraded via the lysosomal compartment upon Bortezomib-imposed inhibition of the proteasome. This conclusion is based on our data showing that impairment of lysosomal acidification by the V-ATPase inhibitor BafA1 prevents ST80/Bortezomib-stimulated I*κ*B*α* degradation, NF-*κ*B activation and BAG3 upregulation. These data provide an explanation for the apparently paradoxical finding that I*κ*B*α* is degraded even when its proteasomal degradation is shut down in the presence of the proteasome inhibitor Bortezomib. I*κ*B*α* has previously been shown to undergo lysosomal degradation under certain conditions. Lee *et al.*^20^ demonstrated that proteasome inhibitors, which block proteasomal degradation of I*κ*B*α*, induce I*κ*B*α* degradation via the lysosome in an IKK-dependent and IKK-independent manner. In addition, nutrient deprivation was described to trigger lysosomal proteolysis of I*κ*B*α* through its binding to heat shock protein 73 (hsc73) and lysosomal glycoprotein 96 (Igp96), a lysosomal membrane receptor.^21^

Our findings have important implications for a better understanding of resistance mechanisms that allow RMS cells to survive proteotoxic stress. By identifying NIK as a key mediator of BAG3 induction and survival upon concomitant inhibition of PQC systems, our findings point to NIK as a possible therapeutic target to overcome acquired resistance to proteotoxic anticancer drugs. Pharmacological inhibitors of NIK have recently been shown to trigger cell death in cancers that depend on constitutive overexpression of NIK for their survival such as Hodgkin lymphoma.^22^ Thus, in future studies it will be interesting to explore whether therapeutic targeting of NF-*κ*B signaling via NIK may open new perspectives to bypass inducible resistance mechanisms to proteotoxic drugs in RMS.

## Materials and Methods

### Cell culture and chemicals

RMS cell lines were obtained from the American Type Culture Collection (Manassas, VA, USA). Cells were maintained in RPMI 1640 or DMEM medium (Life Technologies, Inc., Eggenstein, Germany), supplemented with 10% fetal calf serum (FCS) (Biochrom, Berlin, Germany), 1 mM glutamine (Invitrogen, Karlsruhe, Germany), 1% penicillin/streptomycin (Invitrogen) and 25 mM HEPES (Biochrom). Bortezomib was purchased by Jansen-Cilag (Neuss, Germany); BafA1 from Sigma (Deisenhofen, Germany); SAHA from Selleck Chemicals (Houston, TX, USA). ST80 was synthesized in our lab by previously published procedures.^23, 24^ Chemicals were purchased from Sigma unless otherwise indicated.

### Cell transduction and transfection

For stable RMS NF-*κ*B superrepressor cell lines, we transfected pCFG5-IEGZ vector or pCFG5-IEGZ vector containing I*κ*B*α*-S(32, 36)A (I*κ*B*α*-SR) into PT67 gamma-retroviral producer cells as previously described.^25^ For stable gene knockdown, HEK293T producer cells were transfected with 7.5 *μ*g pGIPZ-shRNAmir vector (Thermo Fisher Scientific, Dreieich, Germany), 12.5 *μ*g pCMV-dR8.91 and 1 *μ*g pMD2.G (Addgene no. 12259) using calcium phosphate transfection as previously described^26^ (non-silencing control: RHS4346, shp65: RHS6934, shp100: RHS0896). Virus-containing supernatant was collected 48 h after transfection and RMS cells were transduced by spin transduction.

For generation of stable GFP-reporter cell lines, we transfected pTRH1-NF-*κ*B EGFP plasmid^27^ in HEK293T producer cells; virus-containing supernatant was collected after 24 h and RMS cells were transduced by spin transduction.

For siRNA interference we reverse-transfected cells with 10 nM siRNA control (cat. #: 4390843) or siRNA against NIK (cat. #: s1786) using Lipofectamine 2000 (Invitrogen) and OptiMEM medium (Invitrogen) according to manufacturer's instruction (Life Technologies, Darmstadt, Germany).

### Determination of NF-*κ*B activation

NF-*κ*B activation was measured by fluorescence-activated cell-sorting (FACS) GFP as previously described^25^ using FACSCanto II (BD Biosciences, Heidelberg, Germany).

### Determination of lysosomal acidification

Lysosomal acidification was determined by staining with 0.05 nM Lysotracker Red (LTR; Invitrogen) and flow cytometry (FACS) according to the manufacturer's instructions.

### Western blot analysis

Western blot analysis was performed as described previously using the following antibodies: mouse anti-phosphorylated I*κ*B*α* (Cell Signaling, Danvers, MA, USA), rabbit anti-I*κ*B*α* (Cell Signaling), rabbit anti-acetylated histone H3 (Millipore, Billerica, MA, USA), rabbit anti-NIK (Cell Signaling), mouse anti-p100/p52 (Millipore), rabbit anti-phosphorylated p65 (Cell Signaling) and rabbit anti-p65 (Abcam, Cambridge, MA, USA). Mouse anti-*α*-Tubulin (Sigma) and mouse anti-GAPDH (HyTest, Turku, Finland) were used as loading controls. Goat anti-mouse IgG and goat anti-rabbit IgG conjugated to horseradish peroxidase (Santa Cruz Biotechnology, Dallas, TX, USA) as secondary antibodies and enhanced chemiluminescence were used for detection (Amersham Bioscience, Freiburg, Germany). Alternatively, infrared dye-labeled secondary antibodies and infrared imaging were used for detection (Odyssey imaging system, LI-COR Bioscience, Bad Homburg, Germany). Representative blots of at least two independent experiments are shown.

### Quantitative reverse transcription PCR (qRT-PCR)

Total RNA was extracted using peqGOLD Total RNA kit from Peqlab Biotechnologie GmbH (Erlangen, Germany) according to the manufacturer's instructions. Three micrograms of total RNA were used to synthesize the corresponding cDNA using RevertAid H Minus First Strand cDNA Synthesis Kit (MBI Fermentas GmbH, St. Leon-Rot, Germany). To quantify gene expression levels, SYBR-Green-based qRT-PCR was performed using the 7900HT fast real-time PCR system from Applied Biosystems (Darmstadt, Germany) according to manufacturer's instructions. Data were normalized on 18S-rRNA expression as reference gene. The following primers (10 pMol/*μ*l) were used: *BAG1-for*: TCACCCACAGCAATGAGAAG; *BAG1-rew*: ATTAACATGACCCGGCAACC; *BAG3-for*: CTCCATTCCGGTGATACACGA; *BAG3-rew*: TGGTGGGTCTGGTACTCCC; *NIK-for*. CCAGCTGCCATCTCTATCATC; *NIK-rew.* AAAAAGTGGGGCTGAACTCT; I*κ*B*α-for.* GTCAAGGAGCTGCAGGAGAT; I*κ*B*α*−*rew*. ATGGCCAAGTGCAGGAAC; *RelB-for*. GCTCTACTTGCTCTGCAGACA; *RelB-rew*. GGCCTGGGAGAAGTCAGC; *TNFα-for*. ACAACCCTCAGACGCCACAT; *TNFα-rew.* TCCTTTCCAGGGGAGAGAGG; *18S-for*. CGCAAATTACCCACTCCCG and *18S-rew*. TTCCAATTACAGGGCCTCGAA. Melting curves were plotted to verify the specificity of the amplified products. All determinations were performed in triplicate. The relative expression of the target gene transcript and reference gene transcript was calculated as ΔΔC_t_.

### Statistical analysis

Statistical significance was assessed by Student's *t*-Test (two-tailed distribution, two-sample, unequal variance).

## Abbreviations

BafA1: Bafilomycin
BAG3: Bcl-2-associated athanogene 3
cIAP: cellular Inhibitor of Apoptosis
FACS: fluorescence-activated cell-sorting
FCS: fetal calf serum
GFP: green fluorescent protein
HDAC6: histone deacetylase 6
HSF1: heat shock factor 1
IKK: I*κ*B kinase
I*κ*B*α*-SR: I*κ*B*α* superrepressor
NF-*κ*B: nuclear factor-kappa B
NIK: NF-*κ*B-inducing kinase
PQC: protein quality control
RMS: rhabdomyosarcoma
SAHA: suberoylanilide hydroxamic acid
TNF: Tumor necrosis factor
TNFR: TNF receptor
TRAF: Tumor necrosis factor receptor-associated factor
UPS: ubiquitin-proteasome system
