# Supplementary material for: NIK is required for NF-κB-mediated induction of BAG3 upon inhibition of constitutive protein degradation pathways
Source: Cell Death Dis. 2015 Mar 12;6(3):e1692–. doi: 10.1038/cddis.2014.584 (PMC4385908; doi:10.1038/cddis.2014.584)
Supplement: Supplementary Information [file cddis2014584x1.pdf]

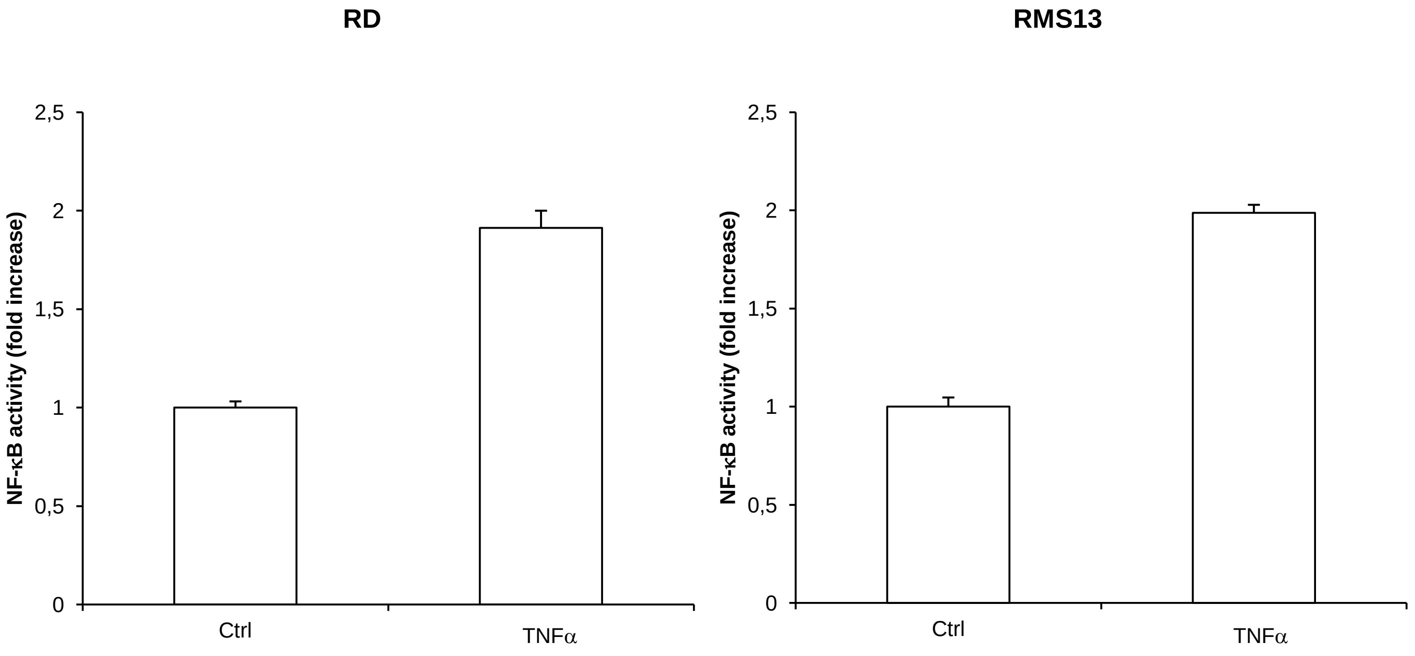
RD
RMS13
NF-κB activity (fold increase)
2,5
2
1,5
1
0,5
0
Ctrl
TNFα

Suppl. Fig. 2

RD **RMS13**

a

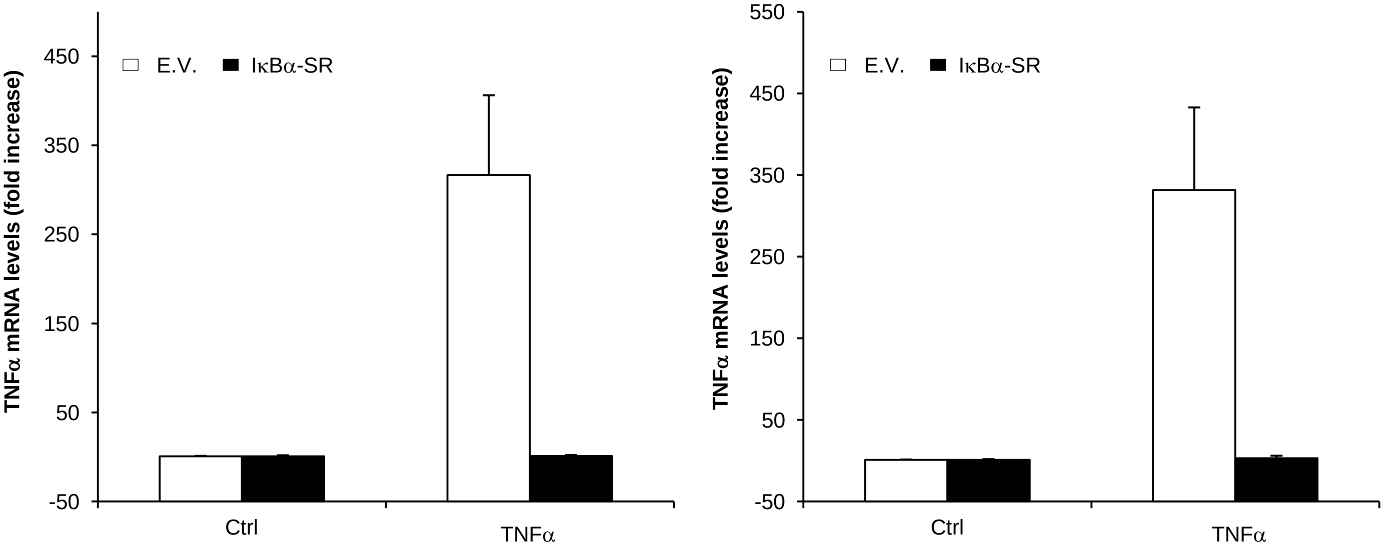

b

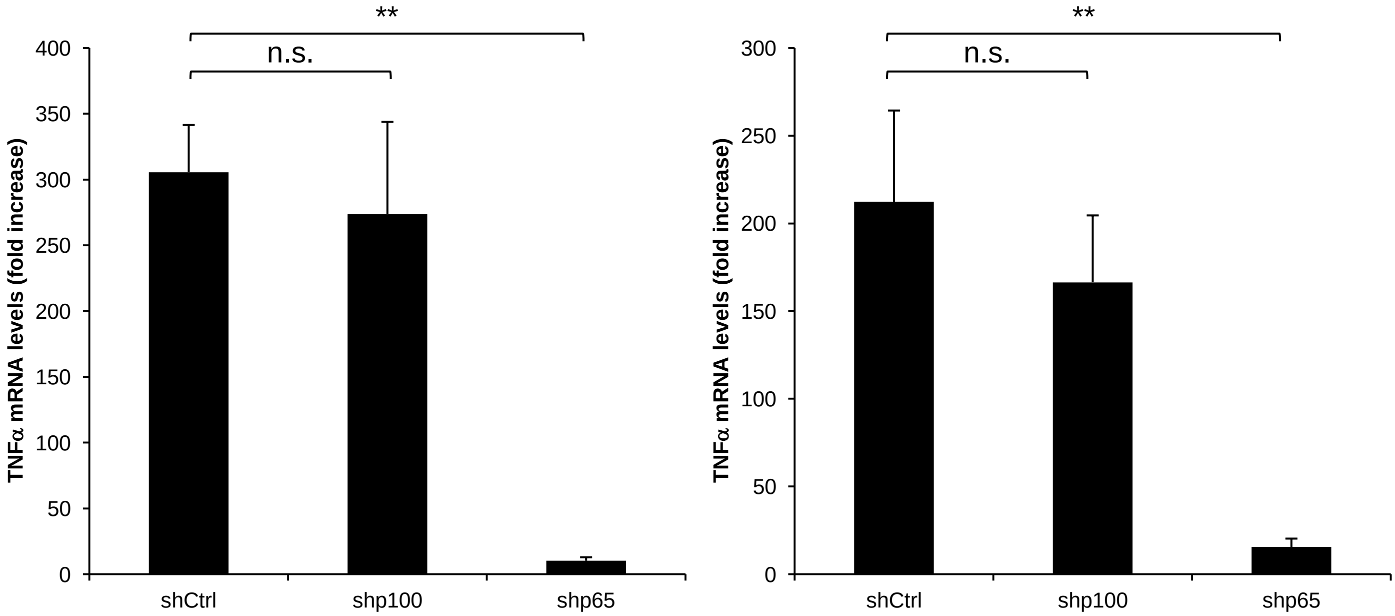

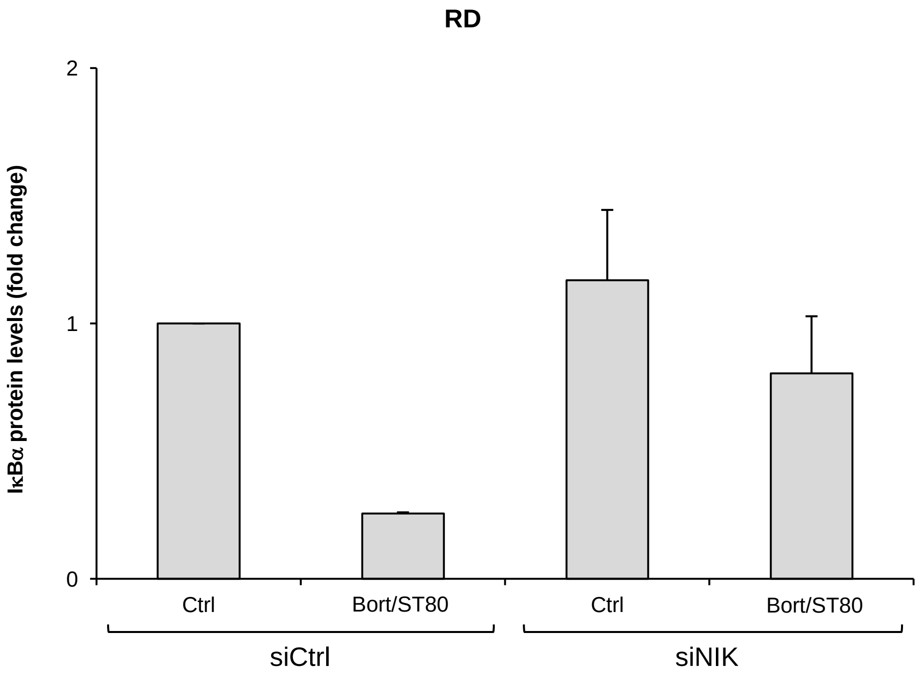
RD
IκBα protein levels (fold change)
2
1
0
Ctrl
Bort/ST80
Ctrl
Bort/ST80
siCtrl
siNIK

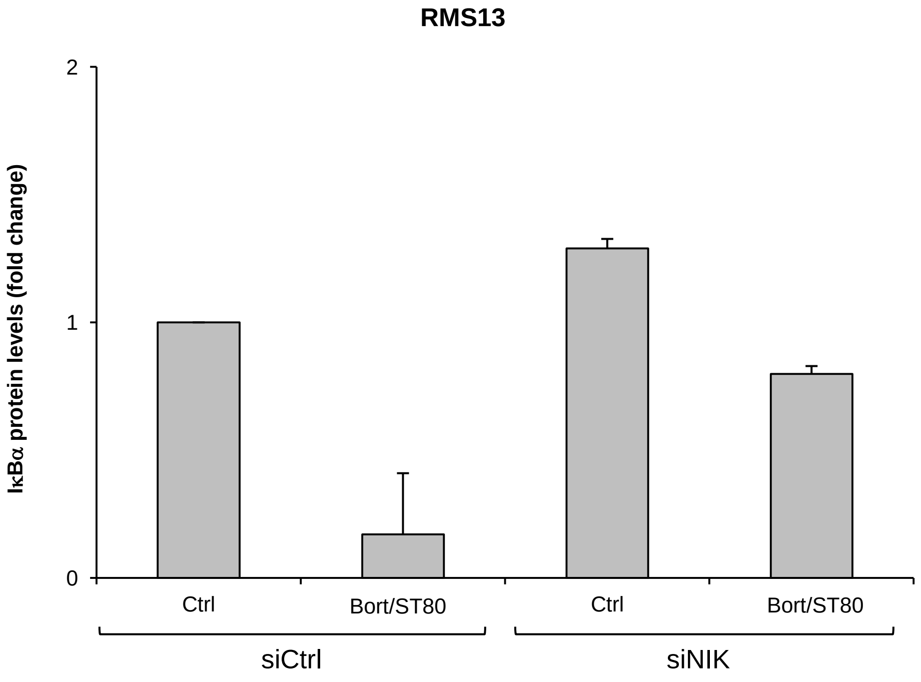
RMS13
IκBα protein levels (fold change)
2
1
0
Ctrl
Bort/ST80
Ctrl
Bort/ST80
siCtrl
siNIK

Suppl. Fig. 4

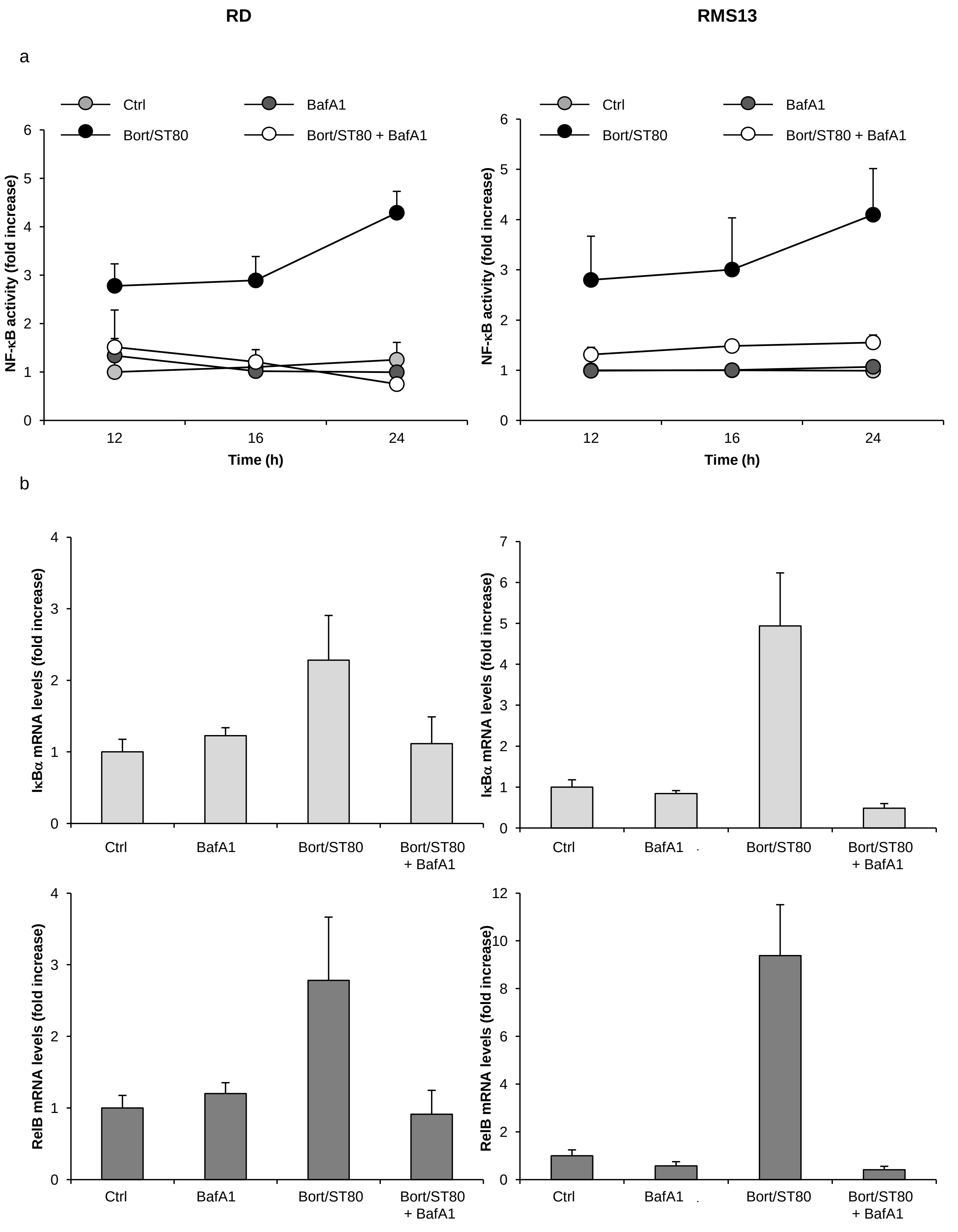

RD
RMS13
a
Ctrl
BafA1
Bort/ST80
Bort/ST80 + BafA1
NF-κB activity (fold increase)
Time (h)
12
16
24
b
IκBα mRNA levels (fold increase)
RelB mRNA levels (fold increase)
Ctrl
BafA1
Bort/ST80
Bort/ST80 + BafA1

Suppl. Fig. 5

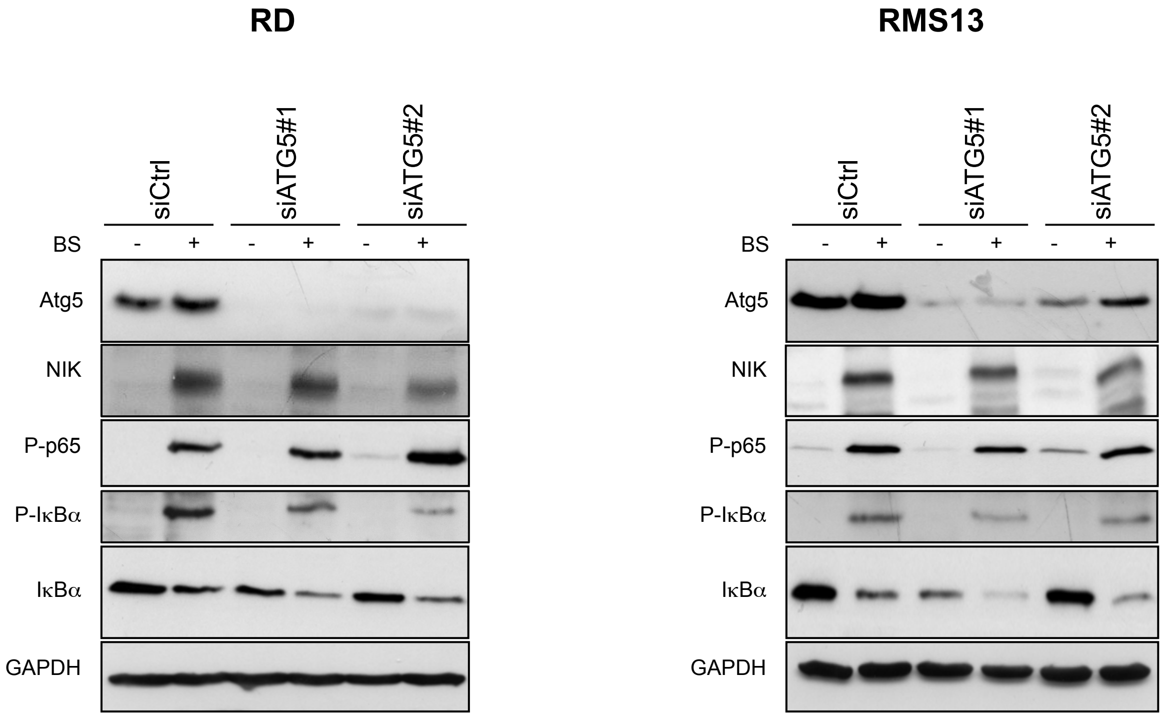
RD
RMS13
siCtrl
siATG5#1
siATG5#2
BS
- + - + - +
Atg5
NIK
P-p65
P-IκBα
IκBα
GAPDH

Supplementary Figure Legends

Suppl. Fig. 1: Genetic inhibition of the canonical NF-κB pathway impairs TNFα induction.

RMS cells stably transfected with pTRH1- NF-κB EGFP plasmid were treated with 10 ng/ml TNFα for 3 hours. NF-κB activation was measured by FACS analysis of FITC florescence.

Suppl. Fig. 2: NF-κB is activated upon ST80/Bortezomib cotreatment.

(a) RMS cells stably transfected with pCFG5-IEGZ vector (E.V.) or pCFG5-IEGZ vector containing IκBα-S(32, 36)A (IκBα-SR) were treated with 10 ng/ml TNFα for 3 hours. TNFα mRNA levels were assessed by RT-PCR.

(b) RMS cells transduced with control vector (shCtrl) or vectors containing shRNA sequence against p65 or p100 were treated with 10 ng/ml TNFα for 3 hours. TNFα mRNA levels were assessed by RT-PCR.

Mean + SD of three independent experiments performed in triplicate are shown; n.s., non significant; **, P<0.01.

Suppl. Fig. 3: NIK accumulation leads to NF-κB activation.

RMS cells stably transfected with pTRH1-NF-κB EGFP plasmid were transiently transfected with siRNA control sequence (siCtrl) or siRNA sequence against NIK (siNIK) and treated for 8 hours with 20 nM (RD) 50 nM (RMS13) Bortezomib and 50 μM ST80 in the presence or absence of 10 nM Bafilomycin A1 (BafA1). Densitometry of IκBα protein levels was evaluated by ImageJ software analysis. Results are presented as $(\text{O.D. I}\kappa\text{B}\alpha/\text{O.D.GAPDH})_{sample}/(\text{O.D. I}\kappa\text{B}\alpha/\text{O.D.GAPDH})_{control}$. Mean + SD of two independent experiments are shown.

Suppl. Fig. 4: Lysosomal activity is required for NF-κB activation.

(a) RMS cells stably transfected with pTRH1- NF-κB EGFP plasmid were treated with 20 nM (RD) or 50 nM (RMS13) Bortezomib and 50 µM ST80 in the presence or absence of 10 nM Bafilomycin A1 (BafA1) at indicated time points. NF-κB activation was measured by FACS analysis of FITC florescence. Data are shown as fold increase of green fluorescence (GFP) compared to untreated cells.

(b) RMS cells were treated with 20 nM (RD) or 50 nM (RMS13) Bortezomib and 50 µM ST80 in the presence or absence of 10 nM Bafilomycin A1 (BafA1) for 12 hours. IκBα (higher panel) and RelB (lower panel) mRNA levels were assessed by RT-PCR. Mean + SD of three independent experiments performed in triplicate are shown.

Suppl. Fig. 5: Effect of ATG5 knockdown on IκBα levels.

RD and RMS13 cells were transiently transfected with two distinct siRNA sequences against ATG5 or control siRNA (siCtrl) and were treated 48 hours after transfection with 20 nM (RD) or 50 nM (RMS13) Bortezomib and 50 µM ST80. Expression levels of ATG5, NIK, P-p65, P-IκBα, IκBα were assessed by Western blot analysis after treatment for 8 hours; GAPDH was used as loading control.

Supplementary materials and methods

For transient knockdown by siRNA cells were reversely transfected with 5 nM SilencerSelect siRNA (Invitrogen) control siRNA (# 4390843) or targeting siRNAs for ATG5 (s18158 and s18160) using Lipofectamine RNAi Max (Invitrogen) and OptiMEM (Life Technologies).
